# Supplementary material for: SNHG5 promotes colorectal cancer cell survival by counteracting STAU1-mediated mRNA destabilization
Source: Nat Commun. 2016 Dec 22;7:13875. doi: 10.1038/ncomms13875 (PMC5192221; doi:10.1038/ncomms13875)
Supplement: Supplementary Information — Supplementary Figures, Supplementary Tables. [file ncomms13875-s1.pdf]

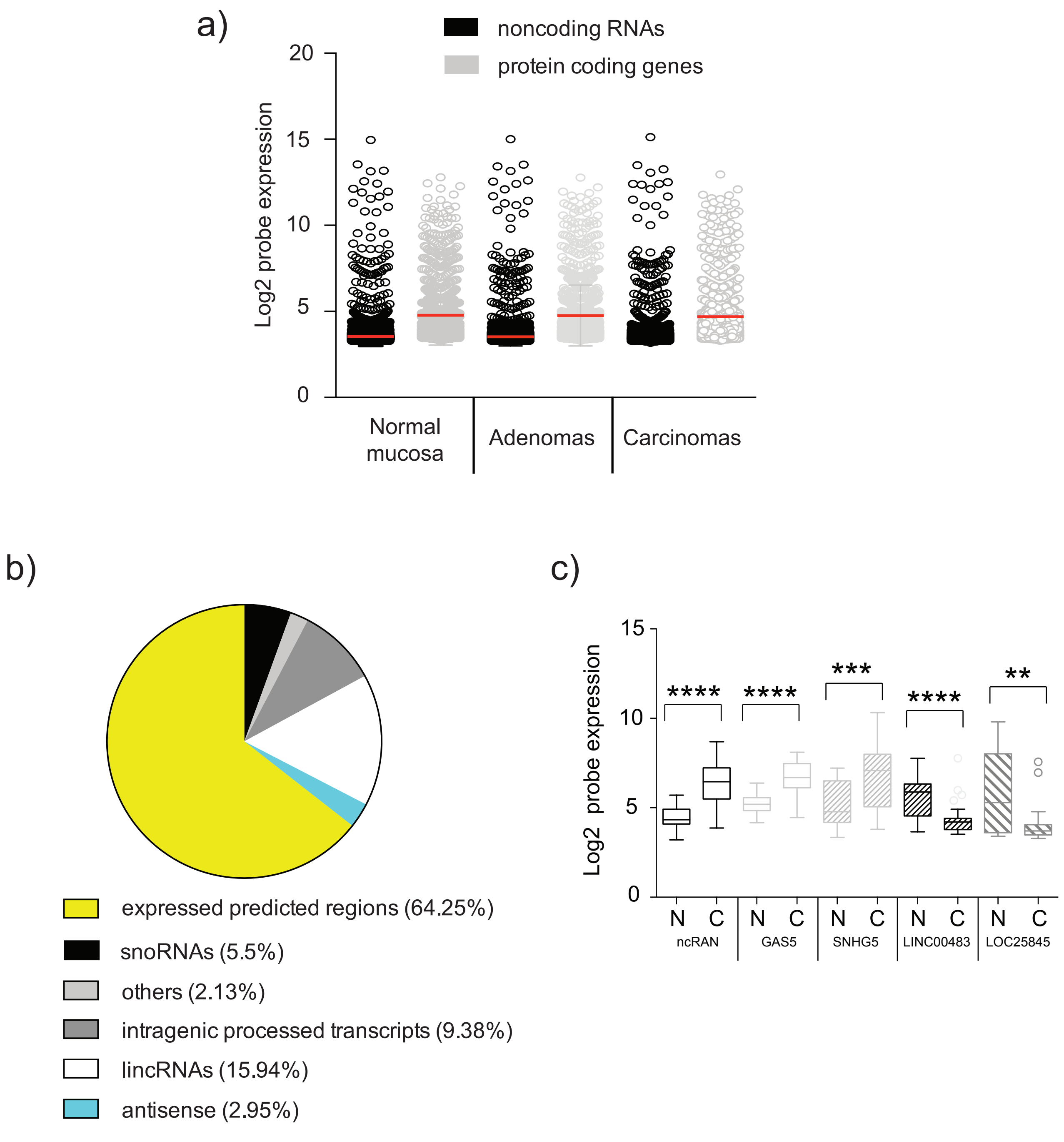

Supplementary Figure 1, Related to Figure 1. SNHG5 is deregulated in Colorectal Carcinomas.

(a) Scatter dot plot representing the overall noncoding RNA and pc genes raw probe signal (Log2) across normal mucosa, adenomas and carcinomas. Each dot represents the average expression of a single transcript. (b) Diagrammatic representation of the different classes of deregulated noncoding RNAs in CRC. (c) Box plot of selected differentially expressed long noncoding RNAs in normal mucosa (N) with respect to carcinomas (C) (*p <0.05, ** p <0.01, *** p <0.001, **** p <0.0001). Mann-Whitney test.

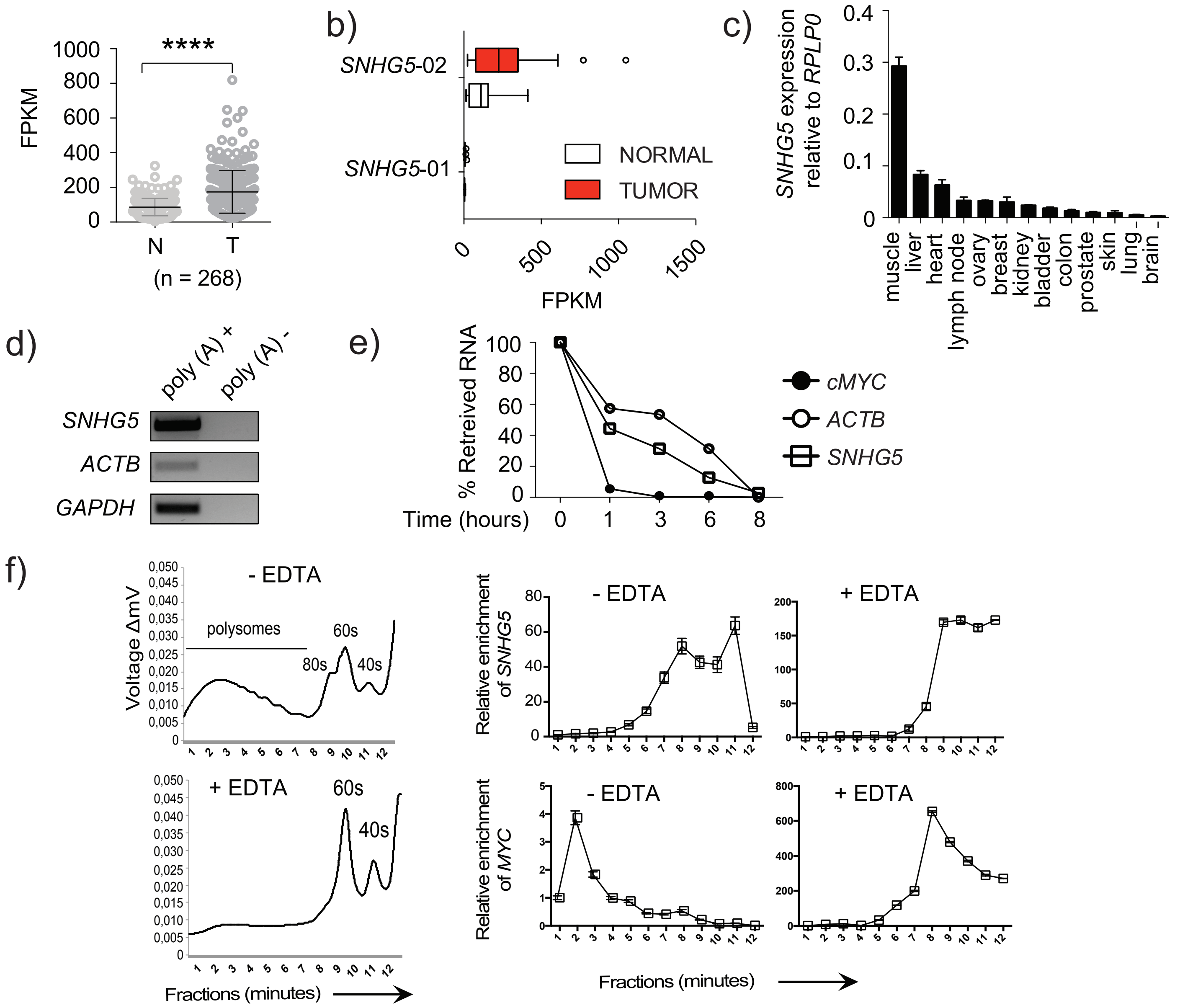

Supplementary Figure 2, Related to Figure 1. SNHG5 is a cytoplasmic long noncoding RNA.

(a) High throughput RNA-sequencing. The expression levels of SNHG5 were profiled in 268 paired clinical specimens, where the RNA was extracted from tumor (C) and normal tissues (N) from the same patient (****p value <0.0001, paired t-test). (b) High throughput RNA-sequencing. Boxplot representing the expression levels of the 2 different SNHG5 isoforms profiled in the 280 CRC patients cohort previously described. (c) qRT-PCR. SNHG5 expression was profiled in a panel of human tissues. Data are normalized to the RPLPO housekeeping gene. Error bars indicating ±s.d. for one representative experiments are included (d) RT-PCR. HCT116 polyadenylated RNA was isolated from the non-polyadenylated fraction and retrotranscribed to cDNA. GAPDH and ACTB primers were used as positive control in the PCR reaction. (e) RT-qPCR. HCT-116 cells were treated with 5 ug/mL of Actinomycin D and the RNA collected at the indicated time points in order to evaluate transcript stability. RNA retrieval at each time point was normalized on the untreated samples. ACTB and MYC were included as controls. (f) Left panel, representative ribosomes traces of HCT116 cell lines treated with 100 ug/ml of cycloheximide. The polysome lysates were fractionated by sucrose centrifugation and collected in 12 1mL tubes. EDTA was added before centrifugation in order to disassemble the polysomes in the negative control sample. Right panel, RT-qPCR. The SNHG5 expression levels are normalized relative to the first fraction collected. MYC expression was included control.

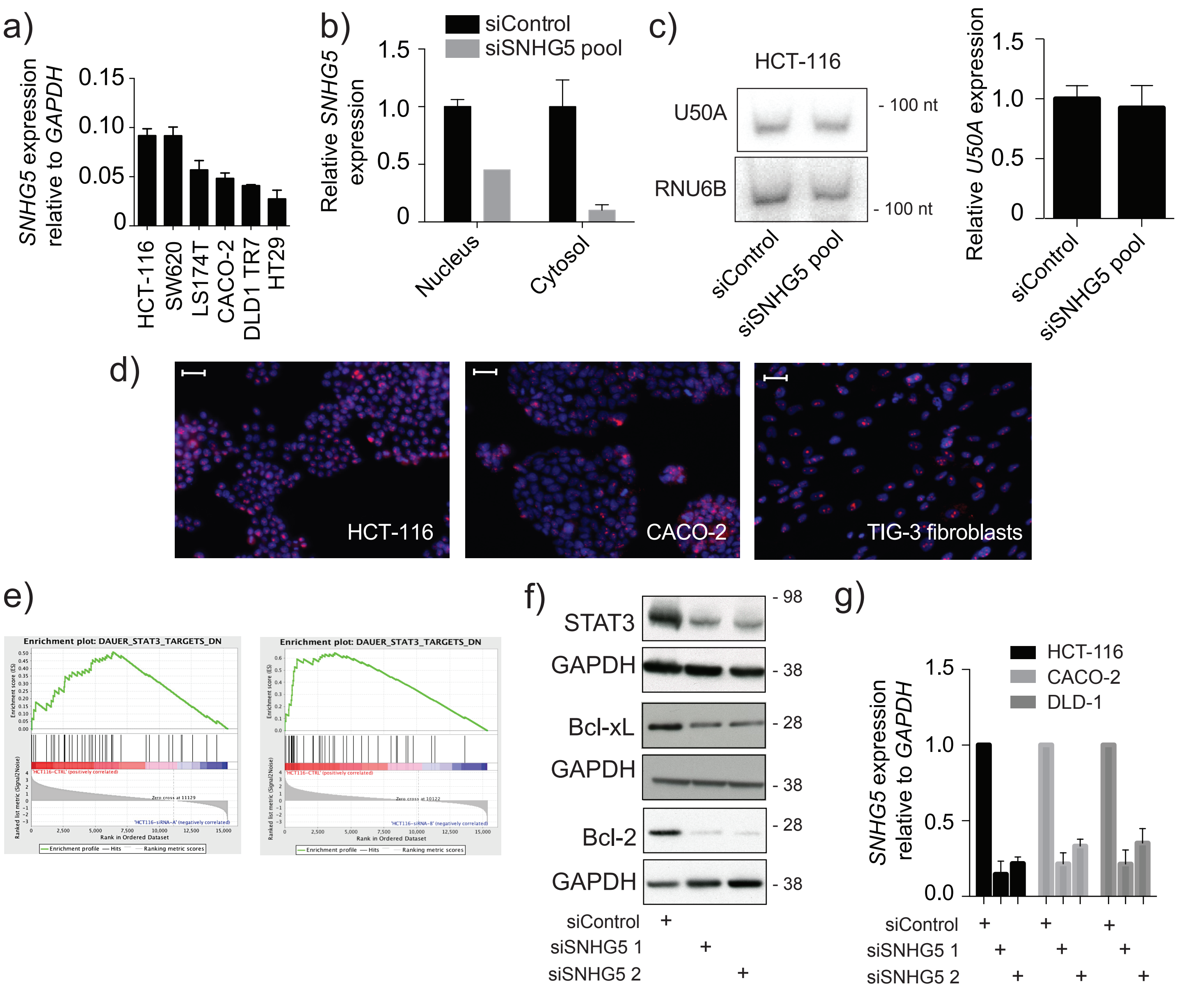

Supplementary Figure 3, Related to Figure 2. SNHG5 regulates CRC cell viability.

(a) qRT-PCR. SNHG5 expression was profiled in a panel of CRC cell lines. Data are normalized to the GAPDH. Error bars indicate ±s.e. for one representative experiment. (b) qRT-PCR. RNA from the nuclear (N) and cytosolic (C) fraction was isolated from HCT116 36 hours after siSNHG5-1/2 pool and siControl transfection. Data were normalized on housekeeping genes and plotted relative to the control sample. Error bars indicating ±s.e. relative for three representative experiments. (c) Northern blot, left panel. HCT-116 cells were transfected with siSNHG5-1 and the RNA collected after 36 hours. U50A expression was detected with a 32P-labelled U50-specific DNA probe. RNU6B expression was used as loading control. Northern blot, right panel. The RNA was extracted from HCT116 cells after subcellular fractionation and Northern blot performed as previously described. (d) RNA-FISH of U50A in red (Cy3) with a nuclear DAPI stain (blue) in HCT116, CACO-2 and TIG3 cell lines. Scale bar = 50 µm. (e) GSEA analysis of gene expression profiles from siControl compared to siSNHG5-1 and siSNHG5-2 transfected HCT-116 cells. (f) Western blot analysis of STAT3, BCL-XL and BCL- 2 levels in HCT-116 cells transfected with siSNHG5 1-2 or siControl. GAPDH is included as loading control. A representative experiment is shown (n=3). (g) CRC cell lines were transfected with 25 nM siSNHG5 1-2 or siControl. Cell number was assessed at the designated time points post transfection. Data normalized to the first time point are shown for the mean of 3 independent experiments.

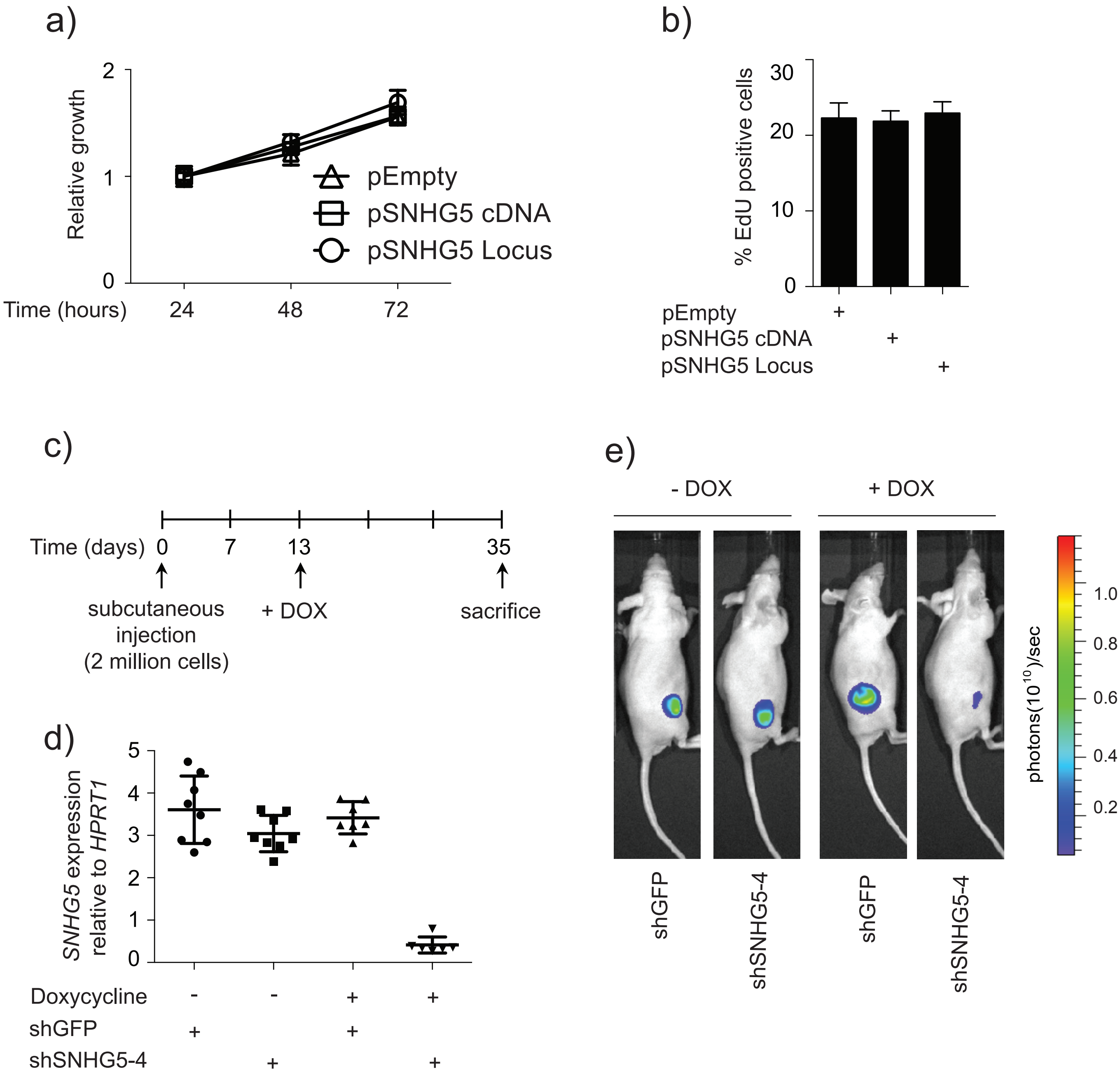

Supplementary Figure 4, Related to Figure 2. SNHG5 regulates CRC cell viability in vitro and in vivo.

(a) 2x105 HT-29 were transfected with 5 ug of indicated plasmids. Proliferation was assessed in each case measuring the absorbance levels of crystal violet solution at 560 nm at the designated time points. Data normalized to the first time point are shown for the mean of 3 independent experiments. Error bars indicating ±s.e. are included. (b) HT-29 cell lines were transfected as previously described. 48 hours after transfection, cells were pulsed with EdU and analyzed by flow cytometry. Data are shown for the mean of three independent experiments. Error bars indicating ±s.e. are included. (c) Timeline for the xenograft experiment. (d) RT-qPCR. RNA was isolated from mice tumors collected at day 35 and SNHG5 expression levels were profiled in the samples. Data were normalized to the HPRT1 housekeeping gene (****$p<0.0001$). Mann-Whitney test. (e) Representative IVIS output of tumor luminescence.

a)

ODD pool EVEN pool

CTTTTACGTCGGCCTTCGCGAGCGTCTGGGCGGGTGGTAGGAACAATGGCGCTGTCTTCA
GTGGCACAGTGGAGCAGCTCTGAAGATGCAAAGATACACGAAAAAACTTCCAGAACATCT
GGGAGAATATTTAATGGAAAATCGCTTGGTTAAAACCTGACACTTTTAACAGTGAACAGC
GTTCTGAGTGTGGACGAGTAGCCAGTGAAGATAATGAATGTCGAATGTGACTGACTAGCA
GCTTCATTTTGAATGAGGGTCGCTGTCTGCCCATTGATAGAGGCCAGATTGTCTTGGAAG
TTCCAAAGTTGCAACGATTTCTGGCTAGTGCCACGAGGTTTACTTGACTGTTGTGTGAAA
AGCTGATAAGAAAACCATCCAGAAAAAAGCTCTTCGTTTTACAAACATGAAAATAAAA CA
TGTAATTTTG

b)

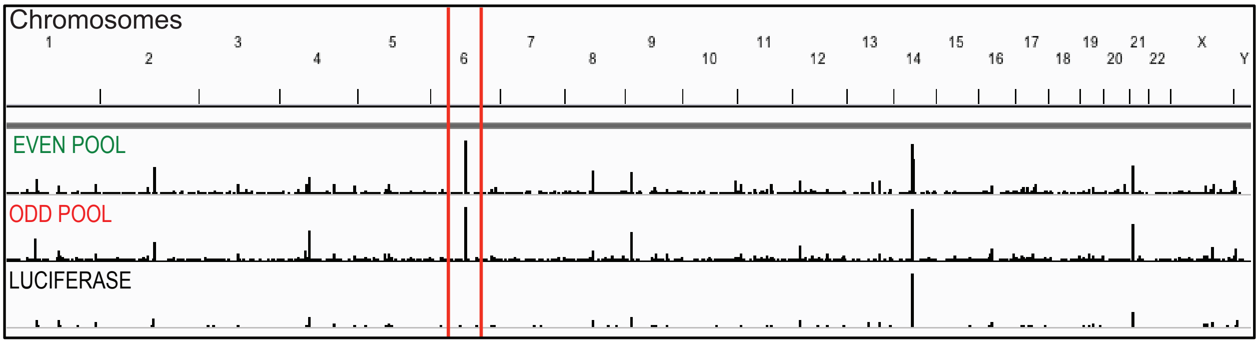

c)

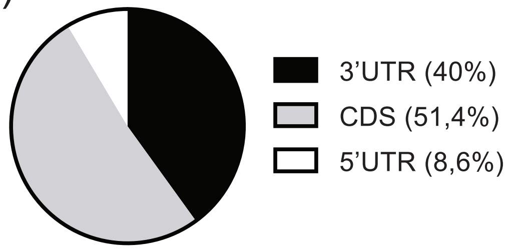

d)

Gene ID	# peaks	enrichment
SPATS2	10	12,72
SNHG5	6	46,95
PITRM1	3	21,03
GLE1	1	16,98
IRX4	1	54,04
ZNF335	2	18,62
CPNE1	1	15,16
SOCS6	1	77,2
KIA00141	1	29,08
CCDC53	1	42,26

e)

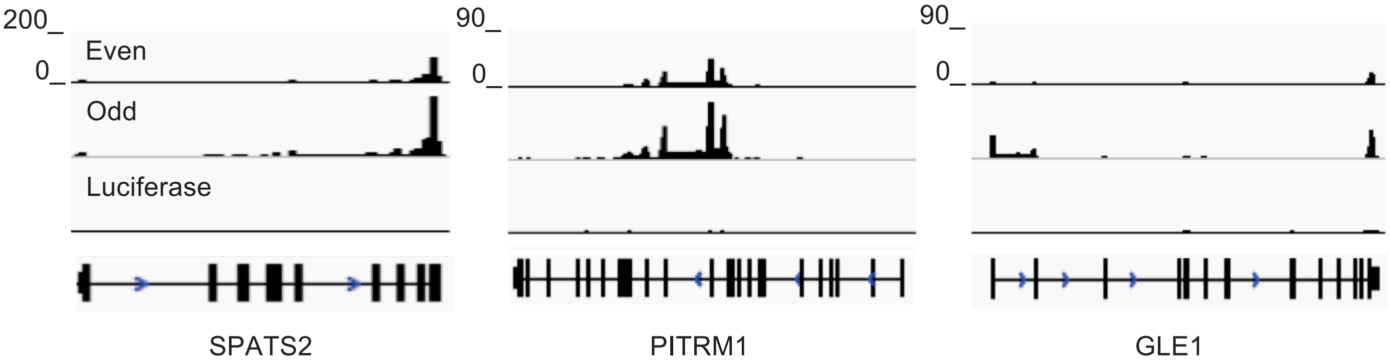

Supplementary Figure 5, Related to Figure 3. RNA Interactome assay identifies SNHG5 direct targets for RNA-RNA interactions.

(a) Schematic representation of the DNA-antisense oligos position on the SNHG5 transcript (Odd pool in bold, Even pool underlined). (b) Graphic output generated by Integrative Genomic Viewer. Each track corresponds to a specific probe pool. In red is indicated the SNHG5 locus. (c) Pie chart summarizes the transcript regions enriched in the SNHG5 pull down. (d) RIA-seq transcript ranking. (e) Graphic output generated by Integrative Genomic Viewer of selected SNHG5 direct interactors. Reads count for each gene is indicated on the y axis.

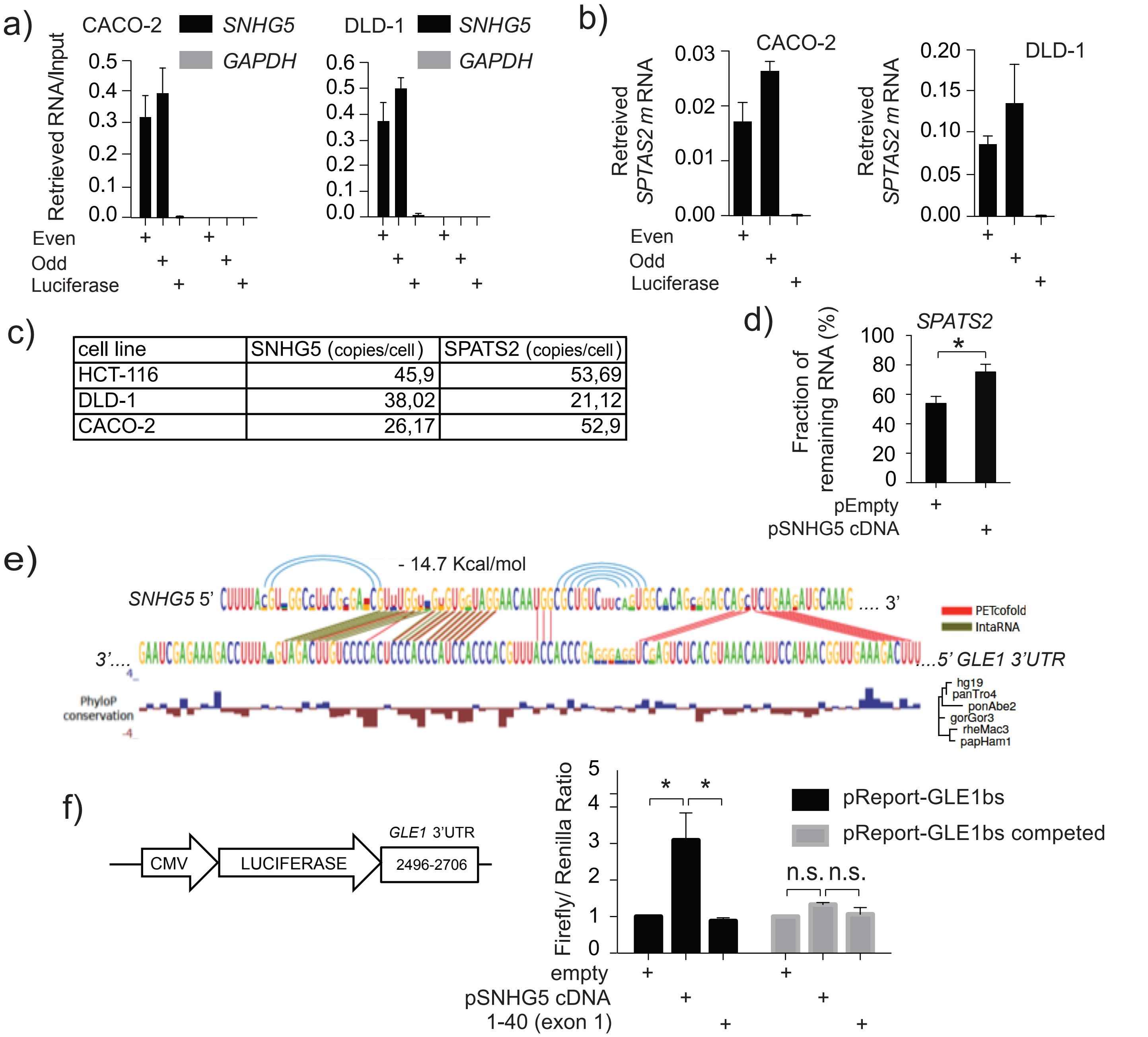

cell line	SNHG5 (copies/cell)	SPATS2 (copies/cell)
HCT-116	45,9	53,69
DLD-1	38,02	21,12
CACO-2	26,17	52,9

Supplementary Figure 6, Related to Figures 4. SPATS2 is a direct target of SNHG5 at post-transcriptional level. (a) qRT-PCR was performed in order to assess the % of SNHG5 and (b) SPATS2 RNA retrieved from CACO-2 and DLD-1 cell lysates following pull down with streptavidin beads. Data was normalized to input (1:100) and GAPDH mRNA included as negative control. Error bars indicate ±s.e. for the mean of three independent experiments. (c) Table summary of the SPATS2 and SNHG5 RNA copy numbers in the indicated CRC cell lines. (d) RT-qPCR. 2x105 HT-29 cells were transfected with the indicated plasmids. 48 hours after transfection the cells were treated with Triptolide at a final concentration of 10 uM for 8 hours and the RNA subsequently extracted. The percentage retrieved SPATS2 mRNA was obtained normalizing to the corresponding expression levels in the untreated cells. (e) Graphical representation of the interaction between SNHG5 exon1 and the GLE1 3'UTR using a combined PETco-fold analysis (red) relative to the evolutionary conservation of the interaction site and IntRNA (green) to map the most thermodynamically stable interaction between the lncRNA and the mRNA. Local PhyloP conservation index is included. (f) Left, schematic representation of the 3'-UTR fragment of GLE1 mRNA cloned in the pMIR-RE-PORT plasmid. Right, luciferase reporter assay performed 48 hours after transfection of 2,4x104 HEK293 cells/well seeded in 96 well plates with the indicated plasmids and a Renilla luciferase transfection control plasmid. In order to compete the SNHG5 binding with the GLE1 3'-UTR, pcDNA 3.1 expressing the entire SPATS2 cDNA was co-transfected with the indicated plasmids. Error bars indicate ±s.e. for 3 independent experiments (*p value <0.05).

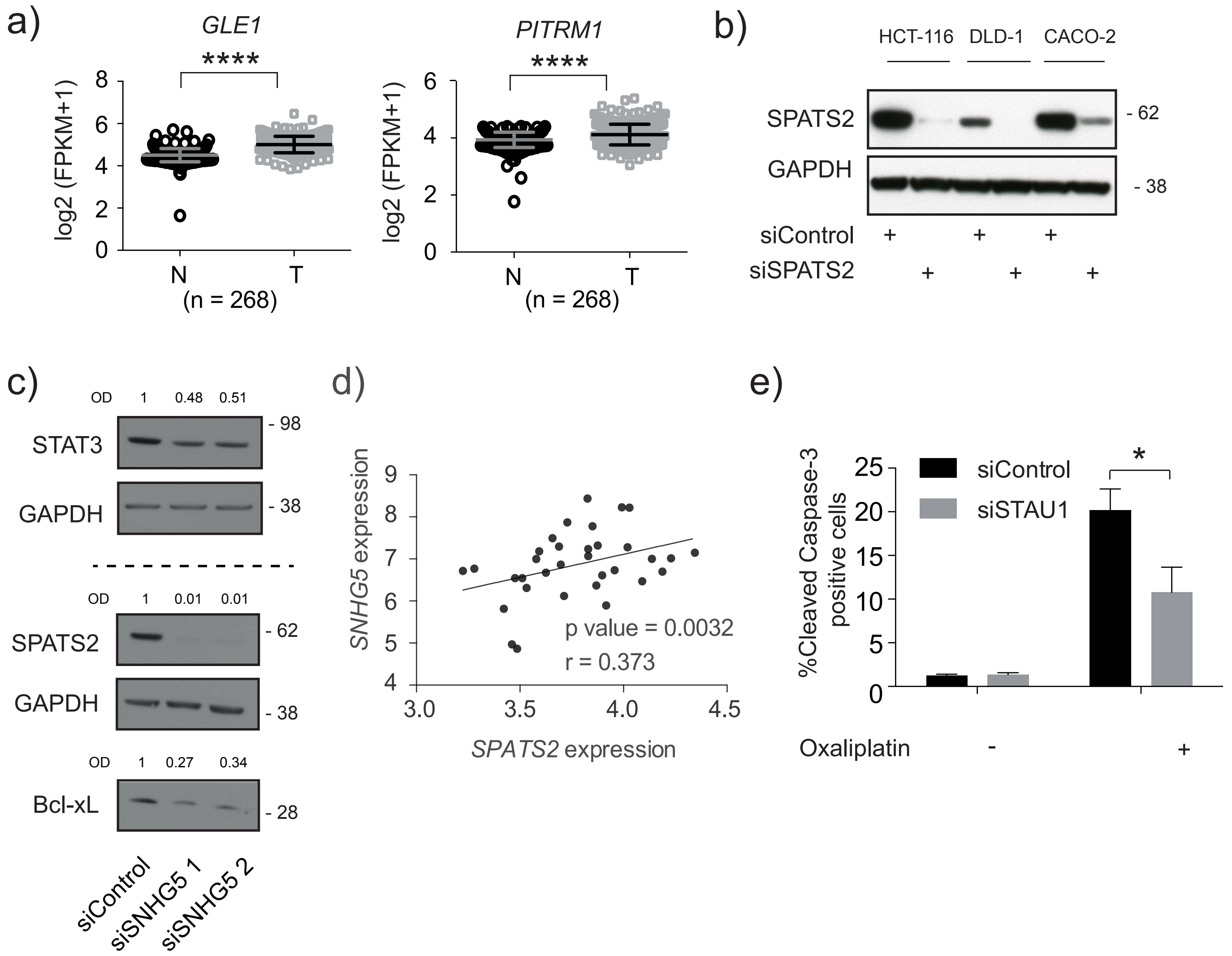

Supplementary Figure 7, Related to Figure 5 and 6. SPATS2 acts downstream SNHG5 regulat-ing survival of CRC cells and SNHG5 impairs the association between STAU1 protein and the SPATS2 mRNA .

(a) Scatter plot. The expression levels of GLE1 and PITRM1 mRNAs ware profiled in paired CRC clinical specimens by RNAseq, where the RNA was extracted from either tumor (C) or normal tissues (N)(****p value <0.0001, Mann-Whitney t-test). (b) Western blot analysis of endogenous SPATS2 levels in CRC cell lines upon transfection with the indicated siRNAs. GAPDH is included as loading control. A representative experiment is shown (n=3). (c) Western blot analysis of endoge-nous SPATS2, STAT3 and Bcl-xL levels in HCT-116 cell lines 48 hours following transfection with the indicated siRNAs. Intensities of protein bands are indicated relative to corresponding loading GAPDH control and normalized relative the non-targeting siControl. A representative experiment is shown (n=3) (d) Scatter plot. Correlation plot of data for SNHG5 and SPATS2 expression levels for 33 adenomas obtained from the RNA sequencing profiling. Correlation coefficient and p value are indicated. Student's t-test was used for statistical analysis. (e) 2x105 HT-29 cells were transfected with indicated siRNAs. 48 hours after transfection the cells were treated with 25 µg/ml oxaliplatin, fixed and stained with Cleaved Caspase-3 antibody for flow cytometry analysis. Data are shown for the mean of three independent experiments. Error bars indicate ±s.e. (*p value <0.05).

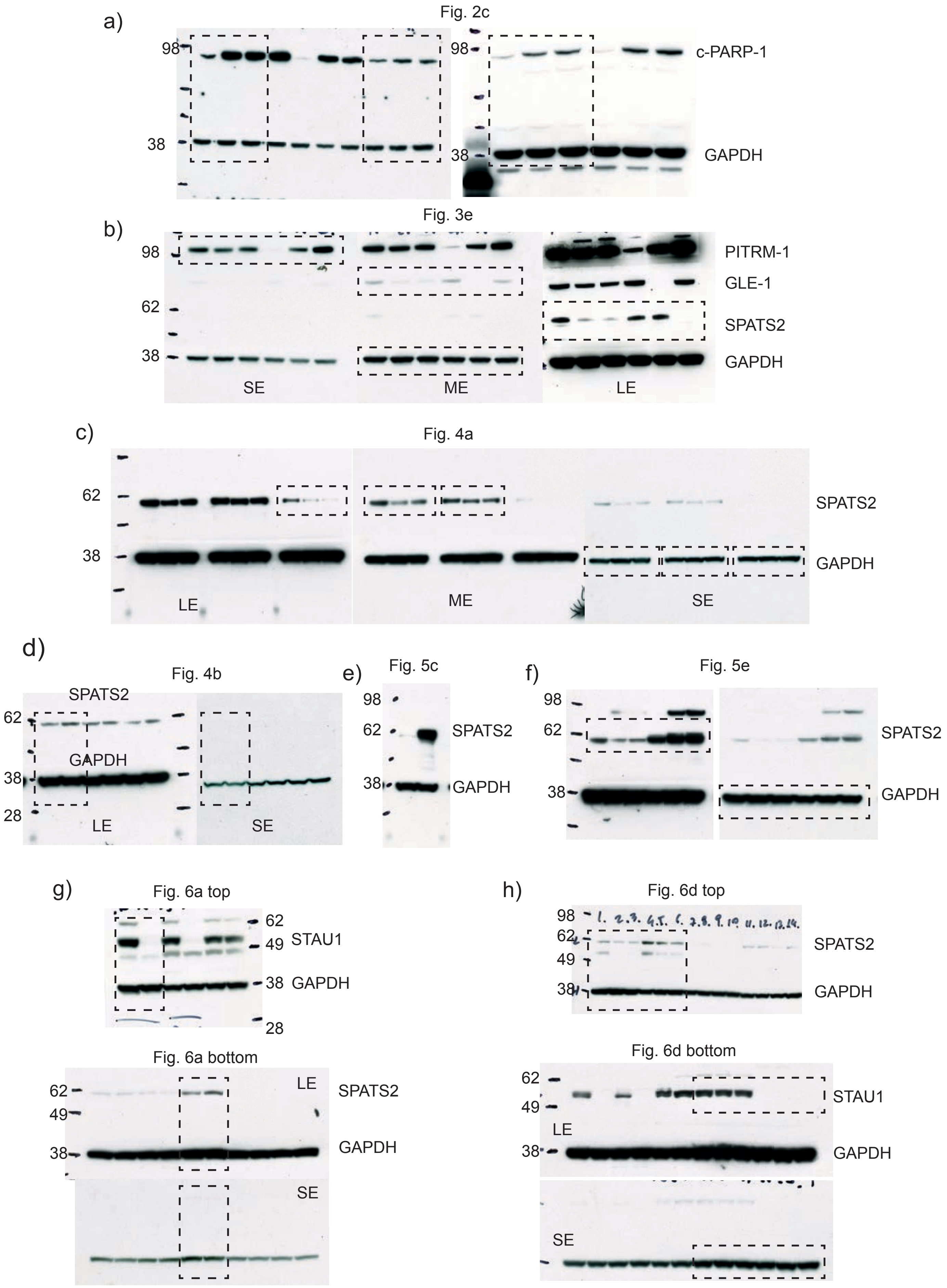

Supplementary Figure 8. Relative to figures 2,3,4,5 and 6.
Short (SE), medium (ME) and long (LE) exposure time are indicated.

Supplementary table 1. List of qPCR and mutagenesis oligos.

qPCR primers	5'->3' sequence
SNHG5 FW	CGCTTGGTTAAAACCTGACACT
SNHG5 RV	CCA AGA CAA TCT GGC CTC TAT C
RPLP0 FW	TTCATTGTGGGAGCAGAC
RPLP0 RV	CAGCAGTTTCTCCAGAGC
HPRT1 FW	GTAATTGGTGGAGATGATCTCTCAACT
HPRT1 RV	TGTTTTGCCAGTGTCAATTATATCTTC
GAPDH FW	ATGGGGAAGGTGAAGGTCG
GAPDH RV	GGGTCATTGATGGCAACAATATC
ARF1 FW1	TTATCTTGGGGAAACCTCAGAA
ARF1 RV1	GTGCTTGTTTGTCGAAATGAAA
H3 FW2	TCTCTTAATCTGCAACCAAGCA
H3 RV2	TGTGGAGGAAAGGAAAGAGCTA
GLE1 FW	ATGGAGAAGAGCTCCAGAGAAG
GLE1 RV	AGCTTCAGGTTGAGAATCTTTG
SPATS2 FW	AAGAGAAGATAAATGCGGTACG
SPATS2 RV	TACTTCACTGGCACTACCTTCC
PITRM1 FW	TGCTGACATCATACATAGCTTC
PITRM1 RV	AGCTAACTGATTTCCCAACTTC
cMYC FW	AGCTGCTTAGACGCTGGATTT
cMYC RV	CGAGGTCATAGTTCCTGTTGGT
ACTB FW	CTCCCCGGGCTGTATTCC
ACTB RV	CCTCTCTTGCTCTGGGCCTC
U50A FW	TATCTGTGATGATCTTATCCCGAACCTGAAC
U50A RV	ATCTCAGAAGCCAGATCCGTAA
Mutagenesis	
0-40 exon FW	CCAGTGTGGTGGAATTCCTTTTGGAACAATGGCG
0-40 exon RV	CGCCATTGTTCCAAAAGGAATTCCACCACACTGG
40-93 exon FW	GTCTGGGCGGGTGGTAGGATACACGAAAAAACTTC
40-93 exon RV	GAAGTTTTTTCGTGTATCCTACCACCCGCCCAGAC
93-172 exon FW	GCTCTGAAGATGCAAAGGTGAACAGCGTTCTGAG
93-172 exon RV	CTCAGAACGCTGTTCACCTTTGCATCTTCAGAGC
172-253 exon FW	CAGACAGCGACCCTCATCTGTTAAAAGTGTCAGGTT
172-253 exon RV	AACCTGACACTTTTAACAGATGAGGGTCGCTGTCTG
253- 322 exon FW	GTGGCACTAGCCATTCAAAATGAAGCTGCTAGTCAGTC
253- 322 exonv RV	GACTGACTAGCAGCTTCATTTTGAATGGCTAGTGCCAC
322-430 exon FW	GTTCCAAAGTTGCAACGATTTCTGGAATTCTGCAGATATCC
322-430 exon RV	GGATATCTGCAGAATTCCAGAAATCGTTGCAACTTTGGAAC

Supplementary table 2.
List of the RNA intaraction assay validation oligos

Primers RIA-seq	5'->3'
ZNF335 FW	GCTAACCCTGCTTCCACATC
ZNF335 RV	GCAGACAGCCTTGGATCTTC
SOCS6 FW	AAGAATTCATCCCTTGGATTAGGTAAC
SOCS6 RV	CAGACTGGAGGTCGTGGAA
IRX4 FW	CCGCCTTCTACTCGCTGA
IRX4 RV	GAGCTGGCTCGTAAGGGTAG
KIAA0141 FW	TCAACTTCCTGGGAACAGAG
KIAA0141 RV	TGTACTGCGCTTTGCTGTAG
PITRM1 FW	ACCGTAAACCAGGTGACATC
PITRM1 RV	TAAATACCTGGCTCCTGTGTC
CPNE1 FW	ATGACTCAGACAAGCTGTTCC
CPNE1 RV	AATTCAAGGCAAATTCATGC
SPATS2 FW	AGAGAAGATAAATGCGGTACG
SPATS2 RV	CTTCCATGAATGCTTGTACTG
PECR FW	TGCTTTGTGGGAGGTAGGTT
PECR RV	GAAAAGCCATCGTGAAGGAG
IRX4 FW	CCGCCTTCTACTCGCTGA
IRX4 RV	GAGCTGGCTCGTAAGGGTAG
GLE1 FW	ACATGCAAGTCTGGGTTATGGT
GLE1 RV	CTAGCCATAGTGTCACCTGAGC
COX15 FW	TGGAGCAGTTATTCTTGGTGGA
COX15 RV	ATTCCTCTTGGCTTGTAGGTGG
CCDC53 FW	GCATGCATAGGGGTACATTTACA
CCDC53 RV	AAGCTTGGTAGTGGACATGTGG

Supplementary table 3.
List of the RNA intarctome assay probe pools, siRNAs and shRNAs.

3' BiotinTEG DNA oligos	
SNHG5-1	CGCGAAGGCCGACGTAAAAG
SNHG5-2	CATTGTTCCTACCACCCGCC
SNHG5-3	CACTGTGCCACTGAAGACAG
SNHG5-4	GTGTATCTTTGCATCTTCAG
SNHG5-5	TGGAAGTTTTTTCGTGTATC
SNHG5-6	TCCATTAAATATTCTCCCAG
SNHG5-7	GTGTCAGGTTTTAACCAAGC
SNHG5-8	TCACTGTTAAAAGTGTCAGG
SNHG5-9	CGTCCACACTCAGAACGCTG
SNHG5-10	CATTATCTTCACTGGCTACT
SNHG5-11	CTAGTCAGTCACATTCGACA
SNHG5-12	GCGACCCTCATTCAAAATGA
SNHG5-13	CTATCAATGGGCAGACAGCG
SNHG5-14	GACAGCGACCCTCATTC
SNHG5-15	TCCAAGACAATCTGGCCTC
SNHG5-16	GGCACTAGCCAGAAATCGTT
SNHG5-17	AGTAAACCTCGTGGCACTAG
SNHG5-18	GGTTTTCTTATCAGCTTTTC
SNHG5-19	AGCTTTTTTCTGGATGGTTT
SNHG5-20	TTCATGTTTGTAAAACGAAG
siRNAs	guide
siControl	Allstar ((1027281, Qiagen)
siSNHG5-1 (targets both isoforms)	UUCGUCCACACUCAGAACG
siSNHG5-2 (SNHG5-2 specific)	CAGCGCCAUUGUUCCUACC
siSTAU1	Silencer s13546 (ThermoFisher)
siPITRM1	SASI_Hs01_00215907 (Sigma)
siSPATS2	SASI_Hs01_001173998 (Sigma)
siGLE1	SASI_Hs0100089249 (Sigma)
shRNAs	guide
shSNHG5	AACCTCGTGGCACTAGCCAGA
shGFP	ATGAACTTCAGGGTCAGCTTG

Supplementary Table 4.

FPKM values for SNHG5 and SPATS2

T/N/A	Stage (I/II/III/I\	ENSG00000203875.5 SNHG5 (FPKM)	ENSG00000123352.12 SPATS2 (FPKM)
N1	-	67,194	12,3493
N2	-	46,8396	5,6903
N3	-	60,1692	10,4675
N4	-	196,658	9,53056
N5	-	91,058	10,3139
N6	-	17,4725	9,34635
N7	-	102,653	8,7523
N8	-	119,358	9,92388
N9	-	77,5841	8,05636
N10	-	127,723	8,80128
N11	-	27,0481	13,7188
N12	-	128,471	8,39966
N13	-	26,2008	9,58686
N14	-	83,1798	11,1858
N15	-	106,211	10,6813
N16	-	25,4842	11,1311
N17	-	83,0968	7,3289
N18	-	113,788	10,3458
N19	-	146,392	10,8743
N20	-	103,728	7,02344
N21	-	58,5109	11,5012
N22	-	98,9285	10,3474
N23	-	13,8233	15,367
N24	-	102,732	12,7451
N25	-	76,2695	12,325
N26	-	93,5359	7,50345
N27	-	62,7014	8,56039
N28	-	114,227	9,9773
N29	-	18,7825	10,5714
N30	-	16,4497	7,50075
N31	-	64,4815	10,922
N32	-	92,0575	9,58194
N33	-	103,585	5,7457
N34	-	23,1615	11,1546
N35	-	22,1305	6,91402
N36	-	73,4674	8,20109
N37	-	91,0742	10,1563
N38	-	59,863	6,95986
N39	-	19,401	13,6981
N40	-	95,2073	9,34763
N41	-	102,339	11,2188
N42	-	121,407	11,5508
N43	-	35,7635	3,61844
N44	-	111,725	10,3691

N45	-	104,318	8,49086
N46	-	80,1779	9,55049
N47	-	25,4327	11,4907
N48	-	89,9943	9,73945
N49	-	23,3897	10,3318
N50	-	131,654	10,5922
N51	-	22,6789	7,97072
N52	-	128,903	6,62122
N53	-	32,0341	6,79483
N54	-	84,0405	11,2275
N55	-	94,2186	10,3344
N56	-	260,456	2,8079
N57	-	134,139	11,9589
N58	-	20,7882	10,2172
N59	-	123,265	9,7437
N60	-	66,4416	8,64227
N61	-	133,099	7,51734
N62	-	57,0392	14,2043
N63	-	111,919	7,64049
N64	-	75,9902	7,6343
N65	-	17,6506	10,6022
N66	-	48,774	11,1939
N67	-	138,069	9,46914
N68	-	160,401	13,4692
N69	-	89,4899	12,5851
N70	-	62,915	8,75218
N71	-	23,6579	8,2457
N72	-	91,9641	9,61681
N73	-	80,0888	6,15184
N74	-	243,62	10,4097
N75	-	216,49	8,47491
N76	-	173,655	8,58494
N77	-	72,4216	1,19413
N78	-	104,5	12,0099
N79	-	170,397	11,7111
N80	-	85,9959	8,38049
N81	-	17,8407	7,8365
N82	-	52,1388	9,33963
N83	-	107,578	8,51923
N84	-	29,303	9,67769
N85	-	64,3128	7,00313
N86	-	157,957	11,3851
N87	-	84,0492	7,04673
N88	-	106,41	6,96709
N89	-	86,9622	10,3214
N90	-	19,7892	7,18567
N91	-	120,102	11,5432
N92	-	85,7937	9,85645
N93	-	122,055	8,64671
N94	-	16,3133	7,5602
N95	-	4,54641	2,28903
N96	-	82,6426	10,3594

N97	-	81,9104	9,19894
N98	-	87,4609	11,542
N99	-	81,45	12,5708
N100	-	116,983	4,57918
N101	-	80,0502	11,8005
N102	-	17,1408	8,09143
N103	-	64,0851	8,30703
N104	-	81,3053	11,9677
N105	-	34,1571	11,6797
N106	-	61,8956	11,2175
N107	-	196,364	5,97651
N108	-	30,481	12,3309
N109	-	80,0519	12,3526
N110	-	58,8775	9,57724
N111	-	23,1141	8,10726
N112	-	115,489	7,09161
N113	-	91,2047	9,32494
N114	-	87,5056	8,97561
N115	-	18,4961	6,43747
N116	-	24,103	10,014
N117	-	83,3333	8,82606
N118	-	25,9628	6,92984
N119	-	126,172	8,29146
N120	-	113,375	12,4411
N121	-	143,36	9,99321
N122	-	83,1078	6,86622
N123	-	100,934	8,9726
N124	-	74,0974	8,24693
N125	-	95,4896	13,2858
N126	-	115,303	8,6338
N127	-	17,1734	6,11445
N128	-	169,738	6,51061
N129	-	23,7171	12,4495
N130	-	96,7179	7,54921
N131	-	164,552	11,2934
N132	-	66,1874	7,29855
N133	-	211,16	11,9038
N134	-	102,962	9,40052
N135	-	20,6887	7,45947
N136	-	89,3373	14,9372
N137	-	102,148	9,78884
N138	-	96,7469	11,9222
N139	-	25,0229	7,3829
N140	-	105,03	8,07409
N141	-	159,094	11,6955
N142	-	110,184	10,0633
N143	-	29,6877	15,7768
N144	-	27,2599	8,17512
N145	-	81,7823	8,25664
N146	-	115,164	8,68062
N147	-	116,044	9,18806
N148	-	84,6355	9,36904

N149	-	85,1701	10,9586
N150	-	70,3623	9,95702
N151	-	151,372	10,3665
N152	-	154,234	8,63946
N153	-	91,8916	8,30831
N154	-	18,3149	13,2182
N155	-	111,664	9,53074
N156	-	170,462	11,5418
N157	-	85,1015	9,51727
N158	-	77,1764	9,29481
N159	-	69,0705	11,1692
N160	-	23,3419	10,1971
N161	-	81,2697	10,7779
N162	-	106,836	12,7019
N163	-	59,2205	6,19768
N164	-	111,365	8,49003
N165	-	92,1868	8,6539
N166	-	120,211	7,62253
N167	-	124,868	8,11479
N168	-	77,6694	11,2186
N169	-	162,203	10,2815
N170	-	22,6495	9,30651
N171	-	67,057	10,103
N172	-	153,234	6,66187
N173	-	17,0042	12,8453
N174	-	16,1412	10,1268
N175	-	162,341	9,63404
N176	-	161,255	8,55622
N177	-	112,499	6,70162
N178	-	107,464	10,0315
N179	-	129,674	4,89648
N180	-	94,8505	9,4595
N181	-	114,015	9,61498
N182	-	63,3677	3,68749
N183	-	245	6,96237
N184	-	90,3803	13,1281
N185	-	86,965	6,88503
N186	-	97,8664	8,59504
N187	-	85,9328	7,67804
N188	-	84,1453	9,12485
N189	-	92,7016	5,3784
N190	-	53,7062	7,49334
N191	-	70,1735	3,84426
N192	-	65,9357	6,12536
N193	-	98,4107	11,5065
N194	-	46,8519	10,3832
N195	-	90,5188	11,6386
N196	-	97,0641	8,50784
N197	-	67,1293	3,70004
N198	-	31,2257	7,75553
N199	-	209,017	17,8913
N200	-	209,333	8,08625

N201	-	105,585	9,58764
N202	-	127,389	11,817
N203	-	147,353	8,35944
N204	-	63,7448	3,49631
N205	-	126,613	6,51525
N206	-	69,305	7,89981
N207	-	163,913	9,62185
N208	-	64,2028	9,59371
N209	-	117,706	10,4591
N210	-	39,8206	7,83018
N211	-	21,8754	9,54576
N212	-	87,4456	8,23786
N213	-	18,167	7,38938
N214	-	111,619	11,7742
N215	-	159,215	7,65484
N216	-	44,6806	8,70106
N217	-	81,4287	5,27372
N218	-	105,914	8,91836
N219	-	83,5999	16,9519
N220	-	101,878	12,117
N221	-	98,8988	9,90911
N222	-	154,15	9,14988
N223	-	100,742	7,86905
N224	-	94,5544	9,62364
N225	-	167,018	13,9351
N226	-	20,4953	11,8384
N227	-	21,9274	12,1784
N228	-	114,438	14,1735
N229	-	46,8696	7,15364
N230	-	119,509	9,96755
N231	-	76,6353	10,3731
N232	-	79,7678	7,50843
N233	-	70,035	11,7825
N234	-	158,526	8,94393
N235	-	85,033	9,70146
N236	-	27,7176	7,3888
N237	-	108,387	11,3131
N238	-	59,8967	6,21443
N239	-	66,9245	9,98289
N240	-	158,226	6,29711
N241	-	24,6682	12,7293
N242	-	72,9021	7,56556
N243	-	78,6126	11,7549
N244	-	82,668	7,14389
N245	-	30,0995	7,42152
N246	-	69,873	10,9113
N247	-	20,3304	11,1366
N248	-	72,7614	7,22703
N249	-	19,6804	6,82681
N250	-	68,1811	9,71041
N251	-	136,613	6,01131
N252	-	23,0261	9,7925

N253	-	116,197	14,858
N254	-	66,9885	7,6731
N255	-	112,853	14,7881
N256	-	60,0454	4,21055
N257	-	66,2193	6,73846
N258	-	184,377	7,80045
N259	-	40,8865	2,84959
N260	-	70,3486	17,6449
N261	-	83,6472	6,6277
N262	-	76,8755	7,39456
N263	-	59,0251	10,0972
N264	-	52,0959	13,2303
N265	-	99,0891	11,1427
N266	-	50,3795	7,16223
N267	-	193,795	14,3408
N268	-	144,326	8,94107
N269	-	99,708	8,34615
N270	-	136,201	9,96159
N271	-	16,5508	10,9611
N272	-	324,928	12,44
N273	-	36,8081	5,04648
N274	-	92,3256	8,63746
N275	-	82,7815	11,3553
N276	-	15,8362	3,92378
N277	-	85,38	7,51244
N278	-	24,4405	9,86134
N279	-	144,705	9,52597
N280	-	84,311	8,173
N281	-	66,4398	12,6344
N282	-	72,876	8,415
N283	-	72,419	9,61103
N284	-	11,115	7,90526
N285	-	33,4832	13,0413
N286	-	15,9014	11,9906
N287	-	58,4561	14,0787
N288	-	78,7349	8,52771
N289	-	82,4632	11,9591
N290	-	64,7531	8,86586
N291	-	60,4825	6,79553
N292	-	66,0146	11,1431
N293	-	19,0023	8,98723
N294	-	23,8697	10,2342
T1	III	49,1813	9,53069
T2	II	131,579	14,6707
T3	I	111,079	14,2259
T4	III	153,181	12,8487
T5	II	56,4587	10,9175
T6	II	35,1074	15,9677
T7	II	187,246	15,3706
T8	III	360,39	12,798
T9	II	151,525	12,1267
T10	I	284,741	14,614

T11	II	129,423	5,9979
T12	II	297,965	14,7956
T13	III	29,007	15,5373
T14	I	66,4941	9,64392
T15	III	194,793	16,7648
T16	I	31,6693	15,0819
T17	III	69,9151	8,14873
T18	II	322,911	12,4894
T19	III	153,479	14,5639
T20	III	137,428	8,28897
T21	II	111,383	11,9935
T22	II	181,568	11,4438
T23	II	91,9721	12,5721
T24	III	141,027	16,2834
T25	I	84,836	12,2265
T26	III	257,283	10,9333
T27	II	216,207	13,4726
T28	I	49,2393	14,4434
T29	II	25,9311	17,2764
T30	II	36,839	10,746
T31	II	310,956	11,1437
A1	Adenoma	96,7897	13,898
T32	I	180,032	16,5316
T33	I	31,9047	13,1142
T34	III	110,426	12,2444
T35	II	189,488	16,7549
T36	III	148,238	11,6854
T37	II	253,505	15,5819
T38	III	25,2988	12,2441
T39	I	169,973	10,7365
T40	II	308,503	12,1508
T41	II	473,192	19,4675
T42	II	206,71	12,0183
T43	I	168,257	10,6202
T44	III	143,537	15,1443
T45	II	234,052	12,4187
T46	I	33,8014	6,97408
T47	I	143,236	11,9812
T48	I	22,7332	13,4992
T49	III	155,415	10,5217
T50	III	49,548	15,7097
T51	II	331,847	10,7051
T52	II	48,5697	12,9039
T53	II	157,798	13,2022
T54	II	341,778	24,1737
T55	III	105,949	9,73129
T56	III	227,072	11,2564
T57	II	25,4424	17,3724
T58	II	819,916	16,5641
T59	II	176,357	23,7164
T60	I	342,008	14,5733
T61	I	112,613	12,5472

T62	III	253,895	13,8611
T63	I	147,532	10,6074
T64	III	60,4518	15,9404
T65	II	213,595	17,6715
T66	III	179,591	17,2622
T67	III	232,301	13,546
T68	II	414,237	11,8131
T69	II	88,4365	11,0837
T70	II	36,3409	17,7043
T71	III	157,045	20,8822
T72	I	449,99	18,5336
T73	I	302,756	8,47453
A2	Adenoma	158,506	13,674
T74	IV	220,169	9,22317
T75	II	57,6212	13,1137
T76	II	133,534	16,7278
T77	II	163,946	10,6537
T78	IV	55,3615	17,2613
T79	III	125,456	10,9735
T80	II	84,1473	20,7723
A3	Adenoma	133,266	13,22
T81	I	153,847	13,2914
T82	I	39,3623	10,8163
T83	II	175,116	13,5974
T84	II	307,947	14,0444
T85	III	118,767	8,1735
T86	II	269,808	13,2576
T87	II	120,191	12,9123
T88	III	59,9602	13,0403
T89	II	397,79	9,82905
T90	III	193,211	12,9237
T91	I	203,892	17,674
T92	II	41,8509	5,92269
T93	I	218,304	13,2509
T94	II	67,2505	14,0276
T95	III	139,408	14,8064
T96	III	76,5755	17,1349
T97	II	165,746	14,193
T98	III	573,986	7,96634
T99	II	146,564	15,694
T100	I	26,1026	15,2888
T101	IV	167,579	9,33652
T102	III	244,981	10,6124
T103	II	76,8373	11,1165
T104	III	73,6657	9,6045
T105	II	200,131	15,192
T106	II	68,338	17,287
T107	I	184,691	20,1053
T108	III	218,45	11,2638
T109	II	51,275	16,5309
T110	II	246,516	18,8791
T111	I	90,7825	10,0153

T112	III	109,481	19,9124
T113	I	32,6918	14,1991
T114	III	26,5358	14,522
T115	II	146,413	13,9124
T116	II	44,4746	10,1427
T117	III	163,461	9,9456
T118	II	144,92	14,431
T119	II	122,908	10,5361
T120	III	214,25	10,1997
T121	I	168,529	21,3913
T122	II	136,81	13,8152
T123	II	125,812	13,5455
T124	II	254,964	15,9251
A4	Adenoma	81,6926	13,6126
A5	Adenoma	101,017	11,3428
T125	III	55,7354	9,78105
T126	II	285,433	26,2881
T127	III	24,281	15,7309
T128	III	131,014	9,37161
T129	III	362,064	10,4768
T130	III	264,344	9,54597
T131	III	138,315	10,2795
T132	II	226,199	25,0591
T133	II	32,216	14,0779
T134	II	232,057	10,7803
T135	IV	251,785	11,1817
T136	II	163,543	12,6056
T137	II	53,1388	13,4931
T138	II	299,016	14,5332
T139	I	267,554	15,9285
T140	III	464,738	17,6309
T141	II	95,9029	16,2104
T142	III	23,2348	11,1077
T143	III	174,845	7,85184
T144	III	166,189	9,54071
T145	II	177,044	13,9472
T146	II	134,243	19,5366
T147	III	111,058	12,7979
T148	I	248,917	8,14418
T149	I	364,173	9,75644
T150	IV	238,697	13,293
T151	II	146,069	13,622
T152	III	33,1162	12,1376
A6	Adenoma	128,408	17,7649
A7	Adenoma	140,852	19,3131
T153	III	234,827	14,2396
T154	I	94,2774	9,90752
T155	IV	126,498	12,5308
T156	II	222,052	14,3407
T157	I	39,3784	14,5111
T158	IV	287,9	16,0618
T159	II	219,783	11,9784

T160	III	31,4928	14,432
T161	II	165,379	8,36419
A8	Adenoma	108,15	8,71061
T162	II	110,771	11,2305
A9	Adenoma	154,146	15,2173
A10	Adenoma	344,726	13,1812
T163	II	153,409	11,804
T164	III	640,959	12,5455
T165	II	23,8566	10,5808
T166	II	296,074	7,81067
T167	III	124,764	11,3027
T168	II	37,6717	19,3134
T169	II	55,9081	17,1215
T170	II	303,573	18,772
T171	I	158,824	14,5225
T172	III	121,016	11,7577
A11	Adenoma	105,261	14,5308
A12	Adenoma	149,575	13,2138
T173	IV	380,937	15,4616
T174	II	236,64	12,0276
A13	Adenoma	127,303	16,5986
T175	III	281,796	18,844
T176	I	105,239	6,40101
A14	Adenoma	298,617	14,9094
T177	II	113,79	12,9012
T178	II	254,341	18,0918
A15	Adenoma	143,945	11,0644
T179	III	94,6485	9,67176
T180	II	62,0055	11,6149
T181	II	261,315	13,6937
T182	III	49,3142	8,80172
T183	II	65,5224	12,3462
T184	III	42,0947	16,5283
T185	I	173,637	14,3396
T186	III	375,702	11,7347
A16	Adenoma	232,478	12,2593
T187	III	80,4829	13,7862
T188	II	396,787	11,4787
T189	III	308,021	15,1474
T190	II	155,85	17,3512
T191	II	252,793	10,1153
T192	II	241,658	16,8588
A17	Adenoma	179,065	11,6157
T193	II	195,295	8,34684
T194	II	27,6138	9,08136
T195	III	235,38	11,1678
T196	III	160,626	13,5417
T197	II	306,288	14,4984
A18	Adenoma	30,3456	10,0128
A19	Adenoma	78,4323	10,5657
T198	III	110,819	9,90187
T199	II	120,282	7,93687

T200	III	249,066	9,43757
T201	II	146,022	7,73129
T202	I	190,498	10,3461
A20	Adenoma	102,922	17,2295
T203	IV	358,996	9,60376
T204	III	421,493	11,4482
T205	III	26,2246	11,4313
T206	I	170,001	9,15103
T207	II	201,349	10,0098
T208	II	405,031	14,5811
T209	III	168,965	12,9291
T210	II	61,7305	10,7605
T211	II	260,928	18,4273
T212	III	47,7192	9,88354
T213	I	45,0217	20,2063
T214	I	172,277	9,94259
T215	III	238,897	13,944
T216	IV	148,6	14,4675
A21	Adenoma	115,657	11,9679
T217	III	178,149	7,45036
T218	III	232,457	19,1871
T219	III	256,299	15,078
T220	III	261,381	11,3297
T221	III	66,4018	11,3785
T222	I	206,559	12,1397
T223	III	375,886	12,0244
T224	II	169,338	17,12
T225	II	186,867	14,5987
T226	III	65,8062	12,5448
T227	II	69,9146	12,525
T228	II	127,768	14,7038
A22	Adenoma	68,4008	12,1149
T229	III	79,0723	13,4593
T230	III	152,831	13,1169
T231	III	45,5752	10,5702
T232	III	103,37	10,537
T233	III	24,5467	13,211
T234	II	141,953	8,7686
T235	II	50,183	11,222
T236	IV	164,046	11,2651
T237	I	231,292	13,0523
T238	III	40,9957	16,5013
T239	II	123,672	10,1392
T240	III	113,32	8,58984
T241	III	224,477	12,1749
T242	II	275,717	11,7873
T243	II	156,754	15,338
A23	Adenoma	296,762	15,3191
T244	II	196,31	5,21121
T245	II	125,408	14,7664
T246	II	90,4548	10,0158
A24	Adenoma	156,008	11,8984

T247	I	165,164	9,90569
T248	II	349,443	7,52118
T249	III	84,5297	13,3287
A25	Adenoma	92,1934	10,127
A26	Adenoma	87,5076	16,0325
A27	Adenoma	104,081	8,34733
T250	IV	76,2591	14,3692
T251	II	291,95	12,1517
A28	Adenoma	126,715	10,9471
T252	II	248,039	10,0552
T253	III	132,419	9,36605
T254	II	65,6847	12,1312
T255	III	249,863	15,9849
T256	II	202,098	11,1218
T257	III	166,69	22,9033
T258	II	59,8069	10,6678
A29	Adenoma	55,2225	9,719
T259	I	213,329	10,7973
T260	II	601,583	7,12074
T261	IV	241,201	13,882
T262	II	365,289	17,6876
T263	II	27,7737	18,4843
T264	II	520,473	10,4388
T265	IV	60,2957	7,53417
T266	III	232,13	6,04766
T267	II	180,155	12,6997
T268	III	39,8109	13,0089
T269	IV	114,286	10,8612
T270	III	33,3895	10,2613
T271	I	647,96	17,333
T272	III	241,313	9,63448
T273	III	195,229	16,7391
T274	II	169,081	11,4221
T275	II	220,004	11,6176
T276	II	33,4731	13,0313
A30	Adenoma	58,4463	14,0988
A31	Adenoma	218,501	13,4235
T277	II	136,697	16,7657
T278	III	111,588	9,57413
A32	Adenoma	92,3729	10,403
T279	IV	122,407	12,8403
A33	Adenoma	28,1235	10,2092
T280	II	76,9431	14,8583
